# An open-label study of pemigatinib in cholangiocarcinoma: final results from FIGHT-202☆

**DOI:** 10.1016/j.esmoop.2024.103488

**Published:** 2024-06-04

**Authors:** A. Vogel, V. Sahai, A. Hollebecque, G.M. Vaccaro, D. Melisi, R.M. Al Rajabi, A.S. Paulson, M.J. Borad, D. Gallinson, A.G. Murphy, D.-Y. Oh, E. Dotan, D.V. Catenacci, E. Van Cutsem, C.F. Lihou, H. Zhen, M.L. Veronese, G.K. Abou-Alfa

**Affiliations:** 1 Hannover Medical School, Hannover, Germany; 2 Toronto General Hospital, Toronto; 3 Princess Margaret Cancer Centre, Toronto, Canada; 4 University of Michigan, Ann Arbor, USA; 5 Gustave Roussy Cancer Center, Paris, France; 6 Providence Cancer Center, Portland, USA; 7 Università degli studi di Verona, Verona, Italy; 8 University of Kansas Medical Center, Kansas City; 9 Baylor University Medical Center, Dallas; 10 Mayo Clinic Cancer Center, Phoenix; 11 Morristown Memorial Hospital, Morristown; 12 Johns Hopkins University School of Medicine, Baltimore, USA; 13 Seoul National University Hospital, Cancer Research Institute, Seoul National University College of Medicine, Integrated Major in Innovative Medical Science, Seoul National University Graduate School, Seoul, Republic of Korea; 14 Fox Chase Cancer Center, Philadelphia; 15 University of Chicago Medicine, Chicago, USA; 16 University Hospitals Gasthuisberg, Leuven & University of Leuven, Leuven, Belgium; 17 Incyte Corporation, Wilmington, USA; 18 Incyte International Biosciences Sàrl, Morges, Switzerland; 19 Memorial Sloan Kettering Cancer Center, New York; 20 Weill Medical College at Cornell University, New York, USA; 21 Trinity College Dublin School of Medicine, Dublin, Ireland

**Keywords:** intrahepatic cholangiocarcinoma, precision medicine, next-generation sequencing, fibroblast growth factor receptor, pemigatinib, targeted therapy

## Abstract

**Background:**

Fibroblast growth factor receptor 2 (*FGFR2*) fusions and rearrangements are clinically actionable genomic alterations in cholangiocarcinoma (CCA). Pemigatinib is a selective, potent, oral inhibitor of FGFR1-3 and demonstrated efficacy in patients with previously treated, advanced/metastatic CCA with *FGFR2* alterations in FIGHT-202 (NCT02924376). We report final outcomes from the extended follow-up period.

**Patients and methods:**

The multicenter, open-label, single-arm, phase II FIGHT-202 study enrolled patients ≥18 years old with previously treated advanced/metastatic CCA with *FGFR2* fusions or rearrangements (cohort A), other *FGF*/*FGFR* alterations (cohort B), or no *FGF*/*FGFR* alterations (cohort C). Patients received once-daily oral pemigatinib 13.5 mg in 21-day cycles (2 weeks on, 1 week off) until disease progression or unacceptable toxicity. The primary endpoint was objective response rate (ORR) in cohort A assessed as per RECIST v1.1 by an independent review committee; secondary endpoints included duration of response (DOR), progression-free survival (PFS), overall survival (OS), and safety.

**Results:**

FIGHT-202 enrolled 147 patients (cohort A, 108; cohort B, 20; cohort C, 17; unconfirmed *FGF*/*FGFR* alterations, 2). By final analysis, 145 (98.6%) had discontinued treatment due to progressive disease (71.4%), withdrawal by patient (8.2%), or adverse events (AEs; 6.8%). Median follow-up was 45.4 months. The ORR in cohort A was 37.0% (95% confidence interval 27.9% to 46.9%); complete and partial responses were observed in 3 and 37 patients, respectively. Median DOR was 9.1 (6.0-14.5) months; median PFS and OS were 7.0 (6.1-10.5) months and 17.5 (14.4-22.9) months, respectively. The most common treatment-emergent AEs (TEAEs) were hyperphosphatemia (58.5%), alopecia (49.7%), and diarrhea (47.6%). Overall, 15 (10.2%) patients experienced TEAEs leading to pemigatinib discontinuation; intestinal obstruction and acute kidney injury (*n* = 2 each) occurred most frequently.

**Conclusions:**

Pemigatinib demonstrated durable response and prolonged OS with manageable AEs in patients with previously treated, advanced/metastatic CCA with *FGFR2* alterations in the extended follow-up period of FIGHT-202.

## Highlights

• FIGHT-202 evaluated pemigatinib in patients with previously treated, advanced/metastatic CCA with *FGFR2* rearrangements.
• Median follow-up was 45.4 months; pemigatinib demonstrated an ORR of 37% and a median DOR of 9.1 months.
• Median PFS and OS were 7.0 and 17.5 months, respectively.
• AEs with pemigatinib treatment were manageable; during extended follow-up, no new safety signals were identified.
• The importance of tumor molecular profiling and pemigatinib efficacy in CCA with *FGFR2* fusions/rearrangements are described.

## Introduction

Cholangiocarcinoma (CCA) accounts for ∼10%-25% of primary hepatic cancers and 3% of gastrointestinal tumors.^1^^,^^2^ In the United States, CCA incidence is increasing.^3^ Older patients, men, and people identifying as Asian/Pacific Islander generally have a higher CCA incidence.^3^ CCA is classified as intrahepatic (iCCA) or extrahepatic (perihilar or distal) based on location. Among CCA tumors, ∼10%-56% are iCCA.^1^^,^^4^^,^^5^ iCCA has high genomic heterogeneity, with 40%-50% of patients with CCA harboring one or more clinically actionable genomic alteration.^6^ Molecular profiling can identify patients most likely to benefit from targeted therapy based on clinically actionable genomic alterations and patterns of co-alterations.^6^, ^7^, ^8^ Fibroblast growth factor receptor 2 (*FGFR2*) fusions and rearrangements have been detected in 1%-13% of patients with iCCA.^7^^,^^9^, ^10^, ^11^ Compared to CCA without *FGFR2* alterations, *FGFR2* fusions are associated with longer overall survival (OS) from diagnosis.^12^ FGFRs regulate several cellular processes, including cell proliferation, survival, migration, and angiogenesis; dysregulation of these pathways drives tumorigenesis.^13^ Therefore, FGFR inhibitors are a rational targeted therapy to disrupt pathogenic FGFR signaling in CCA.^14^

Because of the asymptomatic nature of early-stage disease and nonspecific symptoms in later stages, CCA is often diagnosed in advanced stages when patients are ineligible for curative surgery.^1^^,^^15^ Approximately 65% of patients have unresectable disease, and up to half of them have lymph node metastases at time of diagnosis.^16^^,^^17^ Until recently, gemcitabine plus cisplatin chemotherapy was the first-line standard of care for treatment of unresectable or metastatic CCA.^15^^,^^18^ However, with the European Medicines Agency and United States Food and Drug Administration’s approval of durvalumab in combination with chemotherapy, and pembrolizumab in combination with chemotherapy for locally advanced or metastatic disease, chemoimmunotherapy is now widely accepted as the current standard of care.^19^, ^20^, ^21^, ^22^, ^23^ Modest response rates [∼20% objective response rate (ORR)] and a median survival of ∼11 months are typical with first-line chemotherapy.^24^^,^^25^ The addition of durvalumab to chemotherapy improves ORR to ∼27% and extended median OS to nearly 13 months.^26^ Improvement in OS has also been observed with the addition of pembrolizumab to gemcitabine and cisplatin, resulting in an OS of nearly 13 months.^27^

Despite this recent advance in therapy for unresectable or metastatic CCA,^15^ treatment options that exploit clinically actionable genomic alterations, including *FGFR2* rearrangements, are needed. Pemigatinib is an oral, potent, selective FGFR1-3 inhibitor for treatment of adults with previously treated, unresectable, locally advanced or metastatic CCA with *FGFR2* fusions or other rearrangements.^28^ In the primary analysis of FIGHT-202, a phase II study evaluating the safety and efficacy of pemigatinib in previously treated locally advanced or metastatic CCA, patients with *FGFR2* fusions or rearrangements had an ORR of 35.5% at a median follow-up of 15.4 months.^29^ Here we report final efficacy and safety analyses from the extended follow-up period of the FIGHT-202 study (NCT02924376; EudraCT 2016-002422-36).

## Patients and methods

### Study design

FIGHT-202 was an open-label, single-arm, multicenter, phase II study conducted at 146 sites in the United States, Republic of Korea, UK, France, Italy, Thailand, Germany, Belgium, Israel, Spain, Japan, and Taiwan. The data cut-off date was 8 July 2021. FIGHT-202 consisted of three cohorts based on tumor *FGF*/*FGFR* alteration status: (A) *FGFR2* rearrangements or fusions, (B) other *FGF*/*FGFR* alterations, or (C) no *FGF*/*FGFR* alterations (United States only). Enrollment and initial cohort assignment were permitted based on genomic testing results from a local laboratory. Final cohort assignment for statistical analyses was based on centrally confirmed next-generation sequencing results using the Foundation Medicine clinical trial assay (FoundationOne, Foundation Medicine, Cambridge, MA). FIGHT-202 was carried out as per the International Council for Harmonisation Guideline for Good Clinical Practice, Declaration of Helsinki, and local regulatory requirements. The study protocol was approved by the institutional review board of each site before patient enrollment. All patients provided written informed consent.

### Patients

Eligibility requirements have been published previously.^29^ Briefly, eligible patients were ≥18 years old, had advanced/metastatic or surgically unresectable CCA with radiographically measurable disease as per RECIST v1.1, disease progression after one or more line of prior systemic therapy, documented *FGF*/*FGFR* gene alteration, life expectancy ≥12 weeks, and Eastern Cooperative Oncology Group (ECOG) performance status ≤2. Patients with inadequate hepatic or renal function, history or current evidence of ectopic mineralization or calcification, or current evidence of clinically significant corneal or retinal disorder were ineligible.

### Treatment

All patients self-administered pemigatinib over 21-day cycles (2 weeks on/1 week off) at a starting oral dose of 13.5 mg once daily until documented radiologic disease progression, unacceptable toxicity, consent withdrawal, or physician decision.

### Endpoints and assessments

The primary endpoint was ORR in patients with *FGFR2* fusions or rearrangements (cohort A) as determined by an independent review committee (IRC). ORR was defined as the percentage of patients with complete (CR) or partial responses (PR) as per RECIST v1.1. Disease was assessed by computed tomography or magnetic resonance imaging every 6 weeks through week 12, and every 9 weeks thereafter for all cohorts; patients who discontinued study treatment for reasons other than disease progression were assessed every 9 weeks during follow-up.

Secondary endpoints were ORR in patients with *FGF*/*FGFR* alterations other than *FGFR2* fusions or rearrangements (cohort B) and ORR in patients without *FGF*/*FGFR* alterations (cohort C). Additional secondary endpoints assessed in all cohorts were progression-free survival [PFS; time from first dose to progressive disease (PD) or death], duration of response (DOR; time from the date of CR or PR until PD), disease control rate (DCR; CR + PR + stable disease), and OS (time from first dose to death due to any cause) for all cohorts.

Safety and tolerability were based on the National Cancer Institute Common Terminology Criteria for Adverse Events (CTCAE) version 4.03 and were assessed at screening, during treatment, at the end of treatment, and during follow-up.

Genomic analysis of baseline tumor samples was carried out as previously described.^6^

### Statistical analyses

The efficacy-assessable population included all patients with centrally confirmed *FGF*/*FGFR* alteration status who received one or more dose of pemigatinib. The primary analysis of ORR was carried out in cohort A based on IRC-assessed tumor responses. The Clopper–Pearson method was used to estimate the 95% confidence interval (CI) for ORR. Analyses of ORR and 95% CI estimation in cohorts A and B combined, cohort B, and cohort C, as well as DCR analyses, were carried out in the same way as the analysis of ORR for cohort A. The Kaplan–Meier method was used to assess PFS, DOR, and OS. Exploratory analysis of ORR, PFS, and OS in subgroups based on demographic and baseline clinical characteristics was carried out for cohort A. The safety-assessable population included all enrolled patients who received one or more dose of pemigatinib; safety data were summarized descriptively. Statistical analysis of the effect of co-alterations on OS was carried out using the log-likelihood ratio test and Kaplan–Meier method as described previously.^6^

## Results

### Patients

At final data cut-off, 147 patients were enrolled, including 108 in cohort A (*FGFR2* fusions or rearrangements), 20 in cohort B (other *FGF*/*FGFR* alterations), and 17 in cohort C (no *FGF*/*FGFR* alterations). Two patients had undetermined *FGF*/*FGFR* status as per central review and were excluded from efficacy evaluations. A detailed analysis of *FGFR2* rearrangements (cohort A)^29^ and other genomic alterations (cohorts B and C) has been previously published.^6^ Fifteen and 93 patients assigned to cohort A had *FGFR2* rearrangements and fusions (a subset of rearrangements in which the fusion partner is predicted to be translated in-frame with *FGFR2*),^6^ respectively; 56 unique fusion partner genes were identified. The most common fusion partner was *BICC1* (*n* = 32, 29.6%). In cohort B, the most common *FGF*/*FGFR* alteration was *FRS2* amplification (*n* = 9, 45.0%), followed by *FGF3*, *FGF4*, *FGF19* amplification (*n* = 5, 25.0%), and *FGFR2* C382R point mutation (*n* = 4, 20.0%). In cohort C, the most frequently detected genomic alterations were in *CDKN2A* (*n* = 7, 41.2%), *KRAS* (*n* = 7, 41.2%), and *IDH1* (*n* = 5, 29.4%).

Median (range) age was 59.0 (26-78) years; 101 patients (68.7%) were <65 years old (Table 1). Most patients were women (57.8%), white (70.7%), and enrolled in North America (60.5%). Cohort A included higher percentages of women and patients aged <65 years old compared with cohorts B and C. Most patients (*n* = 132, 89.8%) had iCCA; of these, 107 (99.1%) were in cohort A (Table 1). The most common sites of extrahepatic metastases were the lymph nodes (54.4%) and lung (53.1%). At final data cut-off, 145 patients (98.6%) overall had discontinued treatment (Supplementary Figure S1, available at https://doi.org/10.1016/j.esmoop.2024.103488). The most common reason for pemigatinib discontinuation across cohorts was PD (71.4%), followed by withdrawal by patient (8.2%) and adverse events (AEs; 6.8%). In cohort A, 106 patients (98.1%) discontinued pemigatinib, with PD (71.3%) being the most common primary reason. Median (range) duration of exposure to pemigatinib was 5.9 (0.2-51.1) months overall and was approximately five times longer in cohort A [7.2 (0.2-51.1) months] versus cohorts B [1.4 (0.2-12.9) months] and C [1.2 (0.2-4.7) months]. Treatment information after pemigatinib discontinuation was available for 58 patients (39.5%); of these, 56.9% received one additional line, 19.0% received two lines, and 24.1% received three or more lines of therapy. The most common treatments immediately following pemigatinib discontinuation were chemotherapy (56.9%), futibatinib (17.2%), and immune checkpoint inhibitors (10.3%).

**Table 1:** Patient demographics and baseline clinical characteristics (safety-assessable population)

Parameter	*FGFR2* fusions or rearrangements (*n* = 108)	Other *FGF*/*FGFR* alterations (*n* = 20)	No *FGF*/*FGFR* alterations (*n* = 17)	Total (*N* = 147)^a^
Age, median (range), years	55.5 (26-77)	63.0 (45-78)	65.0 (49-78)	59.0 (26-78)
<65, *n* (%)	83 (76.9)	10 (50.0)	6 (35.3)	101 (68.7)
65-<75, *n* (%)	20 (18.5)	7 (35.0)	8 (47.1)	35 (23.8)
≥75, *n* (%)	5 (4.6)	3 (15.0)	3 (17.6)	11 (7.5)
Sex, *n* (%)				
Female	66 (61.1)	11 (55.0)	7 (41.2)	85 (57.8)
Male	42 (38.9)	9 (45.0)	10 (58.8)	62 (42.2)
Region, *n* (%)				
North America	64 (59.3)	6 (30.0)	17 (100.0)	89 (60.5)
Western Europe	32 (29.6)	3 (15.0)	0	35 (23.8)
Rest of world^b^	12 (11.1)	11 (55.0)	0	23 (15.6)
Race, *n* (%)				
White	79 (73.1)	9 (45.0)	14 (82.4)	104 (70.7)
Asian	12 (11.1)	11 (55.0)	0	23 (15.6)
Black/African American	7 (6.5)	0	1 (5.9)	8 (5.4)
American Indian/Alaska native	0	0	1 (5.9)	1 (0.7)
Other/missing	10 (9.3)	0	1 (5.9)	11 (7.5)
Time since initial diagnosis, median (range), years	1.3 (0.2-11.1)	0.7 (0.2-2.5)	1.0 (0.3-4.3)	1.1 (0.2-11.1)
ECOG performance status, *n* (%)				
0	46 (42.6)	7 (35.0)	6 (35.3)	60 (40.8)
1	57 (52.8)	10 (50.0)	8 (47.1)	76 (51.7)
2	5 (4.6)	3 (15.0)	3 (17.6)	11 (7.5)
Metastatic disease,^c^*n* (%)				
Yes	89 (82.4)	20 (100.0)	16 (94.1)	126 (85.7)
No	16 (14.8)	0	1 (5.9)	18 (12.2)
Missing or not evaluable	3 (2.8)	0	0	3 (2.0)
Prior systemic therapies, *n* (%)				
1	65 (60.2)	12 (60.0)	11 (64.7)	89 (60.5)
2	30 (27.8)	7 (35.0)	2 (11.8)	39 (26.5)
≥3	13 (12.0)	1 (5.0)	4 (23.5)	19 (12.9)
Prior cancer surgery, *n* (%)	38 (35.2)	6 (30.0)	4 (23.5)	48 (32.7)
Prior radiation, *n* (%)	29 (26.9)	3 (15.0)	5 (29.4)	37 (25.2)
CCA location, *n* (%)				
Intrahepatic	107 (99.1)	13 (65.0)	10 (58.8)	132 (89.8)
Extrahepatic	1 (0.9)	4 (20.0)	7 (41.2)	12 (8.2)
Other	0	3 (15.0)	0	3 (2.0)
History of hepatitis, *n* (%)				
Hepatitis B	4 (3.7)	1 (5.0)	0	5 (3.4)
Hepatitis C	1 (0.9)	1 (5.0)	0	2 (1.4)
Sites of disease at baseline, *n* (%)^d^				
Liver	102 (94.4)	17 (85.0)	17 (100.0)	138 (93.9)
Lymph nodes	58 (53.7)	11 (55.0)	10 (58.8)	80 (54.4)
Lung	59 (54.6)	9 (45.0)	10 (58.8)	78 (53.1)
Bone	21 (19.4)	4 (20.0)	2 (11.8)	27 (18.4)
Ascites	8 (7.4)	5 (25.0)	2 (11.8)	15 (10.2)
Pancreas	7 (6.5)	1 (5.0)	2 (11.8)	11 (7.5)

CCA, cholangiocarcinoma; ECOG, Eastern Cooperative Oncology Group; FGF, fibroblast growth factor; FGFR, FGF receptor.

a Total number includes two patients who did not have confirmed *FGF*/*FGFR* status by central laboratory testing and were not assigned to any cohort.

b Rest of the world includes Israel, Japan, Republic of Korea, Taiwan, and Thailand.

c Patients with nonmetastatic disease have no evidence of extrahepatic metastasis. Patients with metastatic disease may have had intrahepatic and extrahepatic metastases.

d Specific sites reported in >5% of patients overall are shown.

### Response to treatment

Overall, median (range) follow-up for the efficacy-assessable population was 45.4 (19.9-53.7) months.

#### Cohort A

Median (range) follow-up for efficacy-assessable patients in cohort A was 42.9 (19.9-52.2) months. ORR (95% CI) based on IRC-assessed confirmed tumor responses was 37.0% (27.9% to 46.9%); three patients (2.8%) achieved CR, and 37 (34.3%) had PR (Table 2). Among 93 patients with *FGFR2* fusions, ORR (95% CI) was 36.6% (26.8% to 47.2%), including two patients (2.2%) with CR and 32 (34.4%) with PR. ORR (95% CI) among 15 patients with *FGFR2* rearrangements was 40.0% (16.3% to 67.7%), including 1 patient (6.7%) with CR and five (33.3%) with PR. In cohort A, outcomes were generally similar across baseline demographic and clinical characteristic subgroups; ORR was numerically higher in patients with ECOG status of 0 versus 1 or 2 (50.0% versus 27.4%) and nonmetastatic (excludes patients with extrahepatic metastases) versus metastatic disease (includes patients with intrahepatic and extrahepatic metastases; 50.0% versus 34.8%), whereas the number of prior therapies did not affect ORR (36.9%, 36.7%, and 38.5%, respectively, for 1, 2, or ≥3 prior therapies; Supplementary Figure S2, available at https://doi.org/10.1016/j.esmoop.2024.103488).

**Table 2:** Efficacy outcomes (efficacy-assessable population)

Parameter	*FGFR2* fusions or rearrangements (*n* = 108)	Other *FGF*/*FGFR* alterations (*n* = 20)	No *FGF*/*FGFR* alterations (*n* = 17)
Duration of follow-up, median (range), months	42.9 (19.9-52.2)	47.5 (43.7-51.1)	51.9 (49.5-53.7)
ORR, *n* (%)	40 (37.0)	0	0
95% CI	27.9-46.9	0-16.8	0-19.5
Best overall response, *n* (%)			
CR	3 (2.8)	0	0
PR	37 (34.3)	0	0
SD	49 (45.4)	8 (40.0)	3 (17.6)
Progressive disease	16 (14.8)	7 (35.0)	11 (64.7)
Not evaluable	3 (2.8)	5 (25.0)	3 (17.6)
Time to response, median (range), months	2.7 (0.7-16.6)	—	—
DOR			
Events, *n* (%)	30 (75.0)	0	0
Censored, *n* (%)	10 (25.0)	0	0
Median (95% CI), months	9.1 (6.0-14.5)	—	—
≥12 months, *n* (%)^a^	12 (30.0)	—	—
Kaplan–Meier estimate (95% CI)			
6 months	67.8 (50.4-80.3)	—	—
12 months	41.2 (24.8-56.8)	—	—
DCR, *n* (%)	89 (82.4)	8 (40.0)	3 (17.6)
95% CI	73.9-89.1	19.1-63.9	3.8-43.4
PFS			
Events, *n* (%)	85 (78.7)	17 (85.0)	15 (88.2)
Censored, *n* (%)	23 (21.3)	3 (15.0)	2 (11.8)
Median (95% CI), months	7.0 (6.1-10.5)	2.1 (1.2-4.9)	1.5 (1.4-1.8)
Kaplan–Meier estimate (95% CI)			
6 months	61.1 (51.0-69.8)	25.3 (8.1-47.1)	6.8 (0.4-26.3)
12 months	32.3 (22.9-42.1)	0 (NE-NE)	0 (NE-NE)
OS			
Deaths, *n* (%)	76 (70.4)	18 (90.0)	15 (88.2)
Censored, *n* (%)	32 (29.6)	2 (10.0)	2 (11.8)
Median (95% CI), months	17.5 (14.4-22.9)	6.7 (2.1-10.6)	4.0 (2.0-4.6)
Kaplan–Meier estimate (95% CI)			
6 months	88.7 (81.0-93.4)	50.8 (26.6-70.7)	26.7 (8.3-49.6)
12 months	67.6 (57.7-75.6)	22.6 (7.0-43.4)	13.3 (2.2-34.6)

CR, complete response; DCR, disease control rate; DOR, duration of response; FGF, fibroblast growth factor; FGFR, FGF receptor; NE, not evaluable; ORR, objective response rate; OS, overall survival; PFS, progression-free survival; PR, partial response; SD, stable disease.

a Calculated as the percentage of patients with DOR ≥12 months among all patients with CR or PR (*n* = 40).

**Figure 1:**
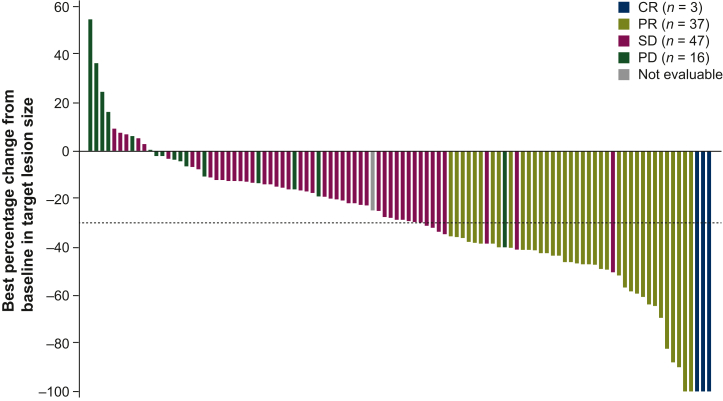
**Best percentage change from baseline in target lesion size based on IRC assessment among efficacy-assessable patients in cohort A (*FGFR2* rearrangements or fusions).** The dashed line indicates criterion for PR (≥30% decrease in sum of target lesion diameters). CR, complete response; FGFR, fibroblast growth factor receptor; IRC, independent review committee; PD, progressive disease; PR, partial response; SD, stable disease.

Median (range) time to response in cohort A was 2.7 (0.7-16.6) months, with a median (95% CI) DOR of 9.1 (6.0-14.5) months (Supplementary Figure S3, available at https://doi.org/10.1016/j.esmoop.2024.103488). Among patients with CR or PR, 12 (30.0%) had DOR ≥12 months. Most patients with DOR ≥12 months had only one line of prior therapy (*n* = 8, 66.7%); four (33.3%) patients had disease in the liver only. Four patients with DOR ≥12 months had *BAP1* co-alterations and none had *TP53* or *PBRM1* co-alterations. DCR (95% CI) in cohort A was 82.4% (73.9% to 89.1%). Among the 104 patients with postbaseline target lesion measurements, 93 had reduction in sum of target lesion diameters, and 48 patients had reductions of >30%. Median (range) best percentage change from baseline in sum of target lesion diameters was −28.4% (−100% to +55%; Figure 1).

#### Cohort B

Median (range) follow-up for efficacy-assessable patients in cohort B was 47.5 (43.7-51.1) months. No objective responses were observed (Table 2). Median (range) best percentage change from baseline in sum of target lesion diameters was 0% (−41% to +91%; Supplementary Figure S4A, available at https://doi.org/10.1016/j.esmoop.2024.103488).

#### Cohort C

Median (range) follow-up for efficacy-assessable patients in cohort C was 51.9 (49.5-53.7) months. No objective responses were observed (Table 2). Median (range) best percentage change from baseline in sum of target lesion diameters was 6.2% (−33% to +74%; Supplementary Figure S4B, available at https://doi.org/10.1016/j.esmoop.2024.103488).

### Progression-free survival and overall survival

**Figure 2:**
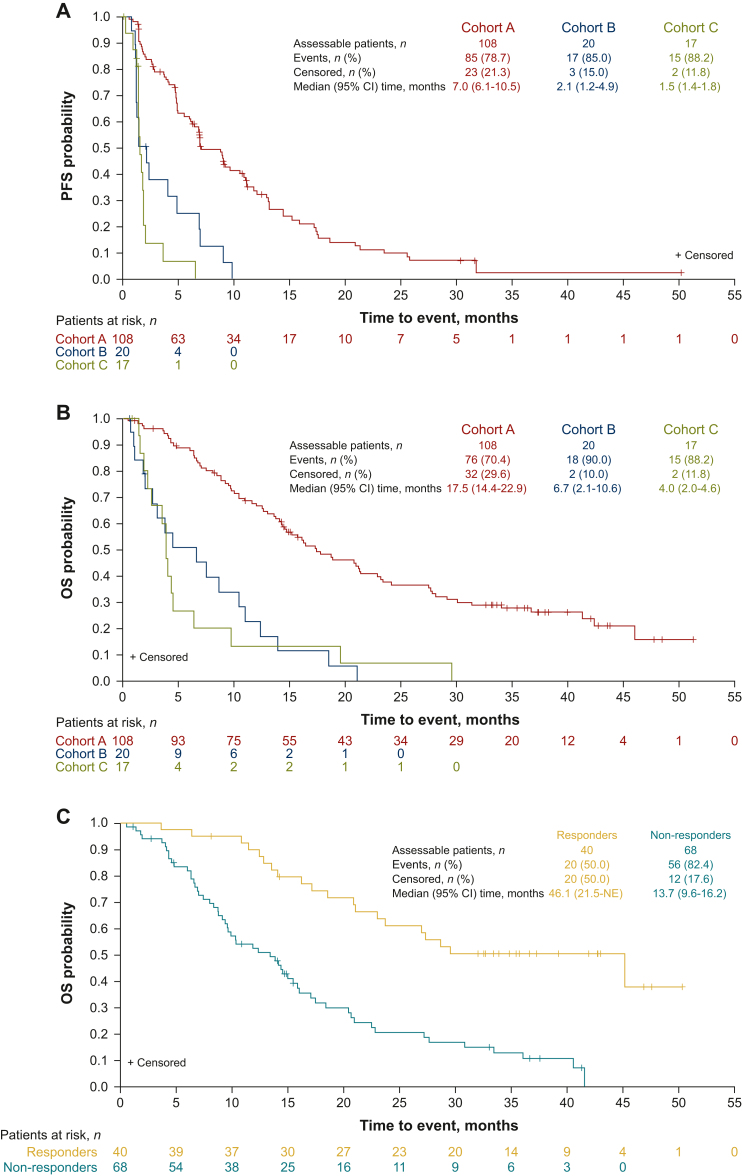
Kaplan–Meier estimates of (A) PFS based on IRC assessment, (B) OS for all cohorts, and (C) OS in cohort A stratified by response (efficacy-assessable population). IRC, independent review committee; NE, not estimable; OS, overall survival; PFS, progression-free survival.

Median (95% CI) PFS based on IRC assessment in cohort A was 7.0 (6.1-10.5) months; Kaplan–Meier estimate of PFS at 12 months was 32.3% (Table 2; Figure 2A). Analysis of PFS among patient subgroups in cohort A revealed that outcomes were generally similar irrespective of patient demographic and baseline clinical characteristics; PFS was numerically shorter among those with metastatic versus nonmetastatic disease (6.9 versus 17.5 months, respectively) and similar between patients with 1, 2, or ≥3 lines of prior therapy (7.0, 8.9, and 6.8 months, respectively; Supplementary Figure S5, available at https://doi.org/10.1016/j.esmoop.2024.103488). PFS for patients with *FGFR2* C382R point mutations (*n* = 4) in cohort B was 1.1, 4.0, 6.9, and 9.0 months, respectively. In cohorts B and C overall, median PFS (2.1 and 1.5 months, respectively) and Kaplan–Meier estimates of PFS at evaluable time points were significantly lower than in cohort A.

At data cut-off, 32 patients (29.6%) in cohort A were alive and censored for survival (Table 2; Figure 2B). Median (95% CI) OS was 17.5 (14.4-22.9) months; Kaplan–Meier estimate of 12-month survival was 67.6%. Median (95% CI) OS was notably longer among responders [46.1 (21.5-not estimable) months] versus non-responders [13.7 (9.6-16.2) months; Figure 2C]. The analysis of OS among patient subgroups in cohort A revealed that outcomes were generally similar across patient demographic and baseline clinical characteristic subgroups (Supplementary Figure S6, available at https://doi.org/10.1016/j.esmoop.2024.103488). Median OS was numerically shorter among patients with ECOG performance status of 1 or 2 versus 0 (14.7 versus 27.7 months, respectively) and for those with versus without metastatic disease (16.2 versus 42.4 months). Patients with co-alterations in *TP53* [hazard ratio (HR) (95% CI) 3.33 (1.48-7.52), *P* = 0.002] and *PBRM1* [HR (95% CI) 2.46 (1.24-4.87), *P* = 0.007] had significantly worse OS compared with patients without the co-alterations (Supplementary Table S1, available at https://doi.org/10.1016/j.esmoop.2024.103488). Two patients each in cohorts B and C (10.0% and 11.8%, respectively) were alive and censored for survival. Median (95% CI) OS was 6.7 (2.1-10.6) months for cohort B and 4.0 (2.0-4.6) months for cohort C; Kaplan–Meier estimates of OS were significantly lower than in cohort A.

### Safety

Overall, all patients experienced one or more treatment-emergent AE (TEAE), and 101 (68.7%) had at least one grade ≥3 TEAE. The most common TEAEs of any grade were hyperphosphatemia (58.5%), alopecia (49.7%), and diarrhea (47.6%). Hypophosphatemia (14.3%), stomatitis (6.8%), and arthralgia (6.1%) were the most common grade ≥3 TEAEs (Supplementary Table S2, available at https://doi.org/10.1016/j.esmoop.2024.103488). Clinically notable TEAEs occurred in 81.6% of patients and included hyperphosphatemia (60.5%), nail toxicity (44.9%), hypophosphatemia (23.8%), and serous retinal detachment (4.8%). Kaplan–Meier estimates of median (95% CI) time to onset of the first occurrence of any hyperphosphatemia or nail toxicity TEAE were 0.49 (0.26-0.69) months and 5.98 (4.80-7.92) months, respectively, and were non-estimable for the first occurrence of hypophosphatemia and serous retinal detachment due to the low incidence of these events. None of these TEAEs led to pemigatinib discontinuation.

Treatment-related TEAEs occurred in 91.8% of patients, and 32.7% experienced grade ≥3 treatment-related TEAEs (Table 3). Overall, the most common treatment-related TEAEs were hyperphosphatemia (53.7%), alopecia (46.3%), and diarrhea (36.1%); the most common grade ≥3 treatment-related TEAEs were hypophosphatemia (8.8%), stomatitis (6.1%), and arthralgia and palmar–plantar erythrodysesthesia syndrome (4.1% each). Serious AEs (SAEs) occurred in 46.3% of patients overall; the most common SAEs included abdominal pain (4.8%), pyrexia (4.8%), and cholangitis (4.1%). Six patients (4.1%) experienced fatal TEAEs, including failure to thrive (*n* = 2), as well as biliary obstruction, cholangitis, sepsis, and pleural effusion (*n* = 1 each); none were deemed related to treatment.

**Table 3:** Treatment-related treatment-emergent adverse events (safety-assessable population)

Events	*FGFR2* fusions or rearrangements (*n* = 108)		Other *FGF*/*FGFR* alterations (*n* = 20)		No *FGF*/*FGFR* alterations (*n* = 17)		Total (*N* = 147)^a^	
	Any grade	Grade ≥3	Any grade	Grade ≥3	Any grade	Grade ≥3	Any grade	Grade ≥3
Any treatment-related TEAE, *n* (%)^b^	102 (94.4)	40 (37.0)	17 (85.0)	6 (30.0)	14 (82.4)	1 (5.9)	135 (91.8)	48 (32.7)
Hyperphosphatemia	55 (50.9)	0	11 (55.0)	0	12 (70.6)	0	79 (53.7)	0
Alopecia	61 (56.5)	0	3 (15.0)	0	2 (11.8)	0	68 (46.3)	0
Diarrhea	44 (40.7)	4 (3.7)	5 (25.0)	0	4 (23.5)	1 (5.9)	53 (36.1)	5 (3.4)
Stomatitis	43 (39.8)	9 (8.3)	4 (20.0)	0	3 (17.6)	0	51 (34.7)	9 (6.1)
Dysgeusia	42 (38.9)	0	3 (15.0)	0	3 (17.6)	0	50 (34.0)	0
Fatigue	38 (35.2)	2 (1.9)	4 (20.0)	0	6 (35.3)	0	48 (32.7)	2 (1.4)
Dry mouth	38 (35.2)	0	2 (10.0)	0	1 (5.9)	0	43 (29.3)	0
Nausea	32 (29.6)	2 (1.9)	2 (10.0)	0	3 (17.6)	0	38 (25.9)	2 (1.4)
Decreased appetite	25 (23.1)	0	5 (25.0)	1 (5.0)	4 (23.5)	0	35 (23.8)	1 (0.7)
Dry eye	33 (30.6)	0	0	0	0	0	34 (23.1)	1 (0.7)
Dry skin	24 (22.2)	1 (0.9)	0	0	0	0	26 (17.7)	1 (0.7)
Arthralgia	21 (19.4)	5 (4.6)	2 (10.0)	1 (5.0)	0	0	23 (15.6)	6 (4.1)
Palmar–plantar erythrodysesthesia syndrome	22 (20.4)	6 (5.6)	1 (5.0)	0	0	0	23 (15.6)	6 (4.1)
Constipation	21 (19.4)	0	1 (5.0)	0	0	0	22 (15.0)	0
Hypophosphatemia	17 (15.7)	11 (10.2)	2 (10.0)	2 (10.0)	0	0	19 (12.9)	13 (8.8)
Vomiting	15 (13.9)	1 (0.9)	1 (5.0)	0	1 (5.9)	0	17 (11.6)	1 (0.7)
Pain in extremity	15 (13.9)	0	0	0	0	0	15 (10.2)	0
Weight decreased	11 (10.2)	1 (0.9)	3 (15.0)	0	0	0	14 (9.5)	1 (0.7)
Hyponatremia	3 (2.8)	1 (0.9)	3 (15.0)	3 (15.0)	2 (11.8)	0	8 (5.4)	4 (2.7)

FGF, fibroblast growth factor; FGFR, FGF receptor; TEAE, treatment-emergent adverse event.

a Total number includes two patients who did not have confirmed *FGF*/*FGFR* status by central laboratory testing and were not assigned to any cohort.

b All any-grade TEAEs occurring in ≥10% and grade ≥3 TEAEs occurring in ≥2% of the total population are shown.

Overall, TEAEs led to pemigatinib dose interruptions, dose reductions, and discontinuations in 42.2%, 13.6%, and 10.2% of patients, respectively. The most frequent TEAEs leading to dose interruptions were stomatitis (8.2%), palmar–plantar erythrodysesthesia syndrome (6.1%), and arthralgia (4.8%). TEAEs leading to dose reductions in more than two patients were arthralgia, palmar–plantar erythrodysesthesia syndrome, and stomatitis [*n* = 5 (3.4%) each]. TEAEs leading to pemigatinib discontinuations in more than one patient were intestinal obstruction and acute kidney injury (*n* = 2 each).

## Discussion

In the final analysis of FIGHT-202, continued benefit of pemigatinib in patients with previously treated advanced or metastatic CCA with *FGFR2* rearrangements or fusions was observed over an extended follow-up period, including a 37% ORR, a median DOR of 9.1 months, and a median PFS and OS of 7.0 and 17.5 months, respectively. No patients with other or without *FGF*/*FGFR* alterations responded to pemigatinib. No new safety concerns were identified; the most common treatment-related TEAE was hyperphosphatemia (54%; all cases were grade 1 or 2).

CCA is typically unresectable at diagnosis,^16^ and mortality rates, primarily driven by iCCA, are increasing.^30^^,^^31^ Standard-of-care first-line treatment for unresectable or metastatic CCA is gemcitabine and cisplatin plus durvalumab in many countries, whereas gemcitabine plus cisplatin chemotherapy remains the standard of care where durvalumab has not yet been approved.^15^^,^^18^^,^^32^ However, many patients do not respond to treatment, and second-line therapies provide only limited benefit.^33^ A meta-analysis of retrospective and phase II studies reporting second-line chemotherapy for bile duct cancers showed a mean response rate of 7.7% and a mean OS of only 7.2 months.^34^ A *post hoc* analysis of FIGHT-202 assessing PFS in patients by prior systemic therapy showed that median PFS in patients with *FGFR2* fusions or rearrangements treated with second-line pemigatinib was 7.0 months.^35^ Patients with prior second-line therapy (chemotherapy, 93%) had a median PFS of 4.2 months, possibly suggesting that second-line targeted therapy may improve outcomes in patients with *FGFR2* fusions or rearrangements over chemotherapy.^35^ In contrast with the historically poor responses to second-line treatments for CCA, we demonstrated the continued clinical benefit of pemigatinib in previously treated CCA with *FGFR2* fusions or rearrangements over an extended follow-up period.

The success of the FGFR inhibitors pemigatinib, erdafitinib, futibatinib, and RLY-4008 in patients with previously treated CCA tumors with *FGFR2* fusions or rearrangements^29^^,^^36^, ^37^, ^38^, ^39^, ^40^ represents a paradigm shift toward personalized medicine.^41^ The European Society for Medical Oncology (ESMO) and United States National Comprehensive Cancer Network (NCCN) treatment guidelines currently suggest biomarker-guided treatments based not only on *FGFR2* alterations but also microsatellite instability-high/mismatch repair-deficient, ERBB2-positive/mutated, *NTRK* fusion-positive, and *BRAF-* and *IDH1-*mutated tumors.^15^^,^^18^ NCCN also suggests biomarker-guided treatments for tumor mutation burden-high and *RET* fusion-positive tumors*,*^15^ and ESMO suggests treatment for patients with *BRCA1/2* or *PALB2* mutations.^18^ Targeted therapies are currently recommended as second-line treatments; however, extending these findings into the first-line setting may improve clinical outcomes in patients with CCA with specific genomic alterations. For example, the ongoing randomized phase III FIGHT-302 clinical study (NCT03656536)^42^ is evaluating first-line pemigatinib versus chemotherapy in patients with CCA with *FGFR2* rearrangements.

The clinical utility of FGFR inhibition in CCA is hampered by primary and secondary resistance to FGFR inhibitors*.* Understanding mechanisms of tumor resistance is therefore critical. In FIGHT-202, patients with co-occurring alterations in one or more tumor suppressor gene had significantly shorter PFS than those without tumor suppressor gene alterations; ORR was not significantly different.^6^ Consistent with previous PFS and ORR findings, *TP53* and *PBRM1* co-alterations were also associated with significantly shorter OS in this final analysis of FIGHT-202.^6^ Patients with co-alterations in *BAP1* also tended to have numerically shorter OS, consistent with the worse PFS previously reported in patients with this co-alteration.^6^ In FIGHT-202, all patients with reductions in tumor size followed by PD (*n* = 8) had developed one or more mutation in the kinase domain of FGFR2 predicted to promote kinase activation or impair pemigatinib binding.^6^ Larger-scale molecular profiling may enhance understanding of alterations that prevent or reverse FGFR inhibition in CCA.

FGFR inhibitors that irreversibly bind to FGFR2 (e.g. futibatinib, RLY-4008) may have greater antitumor activity in CCA with *FGFR2* resistance mutations compared with ATP-competitive inhibitors (e.g. pemigatinib).^14^ However, whether the covalent binding mechanism translates to clinically meaningful longer PFS is unknown. Ongoing phase I/II studies of RLY-4008 will provide more data to address this question.^40^ Preliminary work suggests that sequential treatment of patients with acquired ‘on target’ resistance to ATP-competitive inhibitors with irreversible FGFR2 inhibitors is possible in a subgroup of patients; this concept should be evaluated in future studies.

Although targeting the tumor microenvironment (TME) with immune checkpoint inhibitors has proven to be an effective therapeutic strategy in many solid tumors, these drugs, with the exception of durvalumab^26^ and pembrolizumab,^27^ have been used with limited success in CCA.^1^ Recent TME-based transcriptomic analyses demonstrated that approximately two-thirds of iCCA tumors exhibit an immunologically cold ‘non-inflamed’ TME.^43^ The largest subtype of non-inflamed tumors was significantly enriched in *FGFR2* fusions as well as *BAP1* and *IDH1/2* mutations.^43^ A TOPAZ-1 *post hoc* analysis found that patients with biliary tract cancer with clinically actionable genomic alterations, including *FGFR2* rearrangements, had an OS benefit from first-line durvalumab plus gemcitabine and cisplatin versus chemotherapy alone.^44^ The number of patients with *FGFR2* rearrangements was very small; therefore, whether adding durvalumab to standard chemotherapy benefits patients with *FGFR2* alterations to the same extent as patients without *FGFR2* alterations remains an open question. FGFR inhibition coupled with TME-targeted therapies, such as those that deplete cells contributing to immunosuppressive TME phenotypes, may improve clinical outcomes in specific patient populations.^43^ Further characterization of the TME in *FGFR2-*altered CCA may be warranted to understand the impact of dysregulated FGFR2 signaling on the TME and to identify patients who might benefit most from combination therapies.

Limitations of FIGHT-202 have been discussed previously.^29^ The study design included no active comparator treatment arm. Small numbers in patient subgroups also limit interpretation of efficacy based on demographic and disease characteristic factors.

### Conclusions

This final analysis of FIGHT-202 demonstrated continued durable response, prolonged OS, and manageable AEs in patients with previously treated advanced or metastatic CCA with *FGFR2* fusions or rearrangements, further supporting regulatory approvals of pemigatinib based on this single-arm, phase II study.^28^^,^^45^ These results highlight the need for early molecular testing in CCA. The phase III FIGHT-302 study will further elucidate the role of FGFR inhibitors in biomarker-selected CCA. Routine comprehensive genomic profiling is needed to discover novel actionable *FGFR2* alterations and identify patients who might benefit from FGFR inhibition.
