# Supplementary material for: An open-label study of pemigatinib in cholangiocarcinoma: final results from FIGHT-202
Source: ESMO Open. 2024 Jun 4;9(6):103488. doi: 10.1016/j.esmoop.2024.103488 (PMC11190465; doi:10.1016/j.esmoop.2024.103488)
Supplement: Supplemental Material [file mmc1.docx]

**Supplementary Material**

**An Open-Label Study of Pemigatinib in Cholangiocarcinoma: Final Results From FIGHT-202**

Arndt Vogel, MD, Vaibhav Sahai, MBBS, MS, Antoine Hollebecque, MD, Gina M. Vaccaro, MD, Davide Melisi, MD, PhD, Raed M. Al Rajabi, MD, Andrew S. Paulson, MD, Mitesh J. Borad, MD, David Gallinson, DO, Adrian G. Murphy, MD, Do-Youn Oh, MD, PhD, Efrat Dotan, MD, Daniel V. Catenacci, MD, Eric Van Cutsem, MD, PhD, Christine F. Lihou, BS, Huiling Zhen, PhD, Maria Luisa Veronese, MD, Ghassan K. Abou-Alfa, MD, MBA

# Table S1. Baseline Co-Alterations Associated With OS in Cohort A (*FGFR2* Fusions or Rearrangements)

| **Gene** | **n (%)** | **OS, median (95% CI), mo** | **HR (95% CI)** | ***P-value*** |
| --- | --- | --- | --- | --- |
| *TP53* |  |  |  |  |
| Unaltered | 98 (91.6) | 19.0 (15.0, 24.0) | 3.33  (1.48, 7.52) | 0.002 |
| Altered | 9 (8.4) | 9.8 (6.4, NE) |  |  |
| *PBRM1* |  |  |  |  |
| Unaltered | 97 (90.7) | 19.0 (15.0, 28.0) | 2.46  (1.24, 4.87) | 0.007 |
| Altered | 10 (9.3) | 12.0 (7.6, 17.0) |  |  |
| *LYN* |  |  |  |  |
| Unaltered | 104 (97.2) | 19.0 (15.0, 23.0) | 3.92  (1.20, 12.80) | 0.015 |
| Altered | 3 (2.8) | 6.5 (6.4, NE) |  |  |
| *IDH1* |  |  |  |  |
| Unaltered | 102 (95.3) | 17.0 (14.0, 21.0) | 0.20  (0.03, 1.42) | 0.072 |
| Altered | 5 (4.7) | NE (16, NE) |  |  |
| *TERT* |  |  |  |  |
| Unaltered | 104 (97.2) | 18.0 (15.0, 23.0) | 3.37  (0.80, 14.2) | 0.078 |
| Altered | 3 (2.8) | 8.10 (0.56, NE) |  |  |
| *CDKN2A* |  |  |  |  |
| Unaltered | 86 (80.4) | 19.0 (15.0, 24.0) | 1.61  (0.92, 2.81) | 0.090 |
| Altered | 21 (19.6) | 14.0 (6.8, 28.0) |  |  |
| *SETD2* |  |  |  |  |
| Unaltered | 104 (97.2) | 17.0 (14.0, 23.0) | 0.32  (0.04, 2.31) | 0.233 |
| Altered | 3 (2.8) | — (0.6, —) |  |  |
| *MYC* |  |  |  |  |
| Unaltered | 98 (91.6) | 19.0 (15.0, 23.0) | 1.51  (0.75, 3.04) | 0.247 |
| Altered | 9 (8.4) | 13.0 (9.3, 31.0) |  |  |
| *BAP1* |  |  |  |  |
| Unaltered | 67 (62.6) | 21.0 (15.0, 27.0) | 1.28  (0.80, 2.05) | 0.294 |
| Altered | 40 (37.4) | 16.0 (13.0, 21.0) |  |  |
| *FOXP1* |  |  |  |  |
| Unaltered | 104 (97.2) | 17.0 (14.0, 23.0) | 1.83  (0.57, 5.87) | 0.303 |
| Altered | 3 (2.8) | 19.0 (6.5, NE) |  |  |
| *CDKN2B* |  |  |  |  |
| Unaltered | 93 (86.9) | 19.0 (15.0, 23.0) | 1.38  (0.73, 2.63) | 0.319 |
| Altered | 14 (13.1) | 14.0 (4.9, 29.0) |  |  |
| *FANCG* |  |  |  |  |
| Unaltered | 104 (97.2) | 18.0 (15.0, 23.0) | 1.76  (0.55, 5.63) | 0.331 |
| Altered | 3 (2.8) | 11.0 (7.6, NE) |  |  |
| *MCL1* |  |  |  |  |
| Unaltered | 99 (92.5) | 17.0 (14.0, 21.0) | 0.65  (0.26, 1.62) | 0.348 |
| Altered | 8 (7.5) | 28.0 (13.0, NE) |  |  |
| *LRP1B* |  |  |  |  |
| Unaltered | 104 (97.2) | 18.0 (15.0, 23.0) | 1.72 (0.54, 5.49) | 0.354 |
| Altered | 3 (2.8) | 11.0 (11.0, NE) |  |  |
| *KIAA1217* |  |  |  |  |
| Unaltered | 103 (96.3) | 17.0 (14.0, 23.0) | 0.55 (0.13, 2.24) | 0.398 |
| Altered | 4 (3.7) | 30.0 (6.4, NE) |  |  |
| *PTEN* |  |  |  |  |
| Unaltered | 102 (95.3) | 17.0 (14.0, 23.0) | 0.70  (0.22, 2.23) | 0.548 |
| Altered | 5 (4.7) | 19.0 (8.1, NE) |  |  |
| *AHCYL1* |  |  |  |  |
| Unaltered | 104 (97.2) | 17.0 (14.0, 23.0) | 1.38  (0.43, 4.40) | 0.588 |
| Altered | 3 (2.8) | 21.0 (11.0, NE) |  |  |

FMI, Foundation Medicine, Inc.; HR, hazard ratio; NE, not estimable; OS, overall survival.

Genes with ≥3 altered samples and log-rank *P* <0.6 shown.

Table S2. Treatment-Emergent Adverse Events (Safety-Evaluable Population)

| Events | ***FGFR2* fusions or rearrangements (n=108)** | | **Other *FGF/FGFR* alterations (n=20)** | | **No *FGF/FGFR* alterations (n=17)** | | **Total (N=147)^a^** | |
| --- | --- | --- | --- | --- | --- | --- | --- | --- |
|  | **Any Grade** | **Grade ≥3** | **Any Grade** | **Grade ≥3** | **Any Grade** | **Grade ≥3** | **Any Grade** | **Grade ≥3** |
| Any TEAE, n (%)^b^ | 108 (100.0) | 72 (66.7) | 20 (100.0) | 15 (75.0) | 17 (100.0) | 13 (76.5) | 147 (100.0) | 101 (68.7) |
| Hyperphosphatemia | 60 (55.6) | 0 | 13 (65.0) | 0 | 12 (70.6) | 0 | 86 (58.5) | 0 |
| Alopecia | 64 (59.3) | 0 | 4 (20.0) | 0 | 3 (17.6) | 0 | 73 (49.7) | 0 |
| Diarrhea | 58 (53.7) | 4 (3.7) | 5 (25.0) | 0 | 6 (35.3) | 1 (5.9) | 70 (47.6) | 5 (3.4) |
| Fatigue | 50 (46.3) | 5 (4.6) | 5 (25.0) | 0 | 9 (52.9) | 3 (17.6) | 64 (43.5) | 8 (5.4) |
| Nausea | 46 (42.6) | 3 (2.8) | 7 (35.0) | 0 | 7 (41.2) | 0 | 61 (41.5) | 3 (2.0) |
| Stomatitis | 46 (42.6) | 10 (9.3) | 6 (30.0) | 0 | 3 (17.6) | 0 | 56 (38.1) | 10 (6.8) |
| Constipation | 46 (42.6) | 1 (0.9) | 5 (25.0) | 0 | 2 (11.8) | 0 | 54 (36.7) | 1 (0.7) |
| Dysgeusia | 45 (41.7) | 0 | 3 (15.0) | 0 | 3 (17.6) | 0 | 53 (36.1) | 0 |
| Decreased appetite | 34 (31.5) | 1 (0.9) | 8 (40.0) | 1 (5.0) | 7 (41.2) | 1 (5.9) | 50 (34.0) | 3 (2.0) |
| Dry mouth | 42 (38.9) | 0 | 5 (25.0) | 0 | 1 (5.9) | 0 | 50 (34.0) | 0 |
| Arthralgia | 37 (34.3) | 7 (6.5) | 5 (25.0) | 2 (10.0) | 2 (11.8) | 0 | 44 (29.9) | 9 (6.1) |
| Vomiting | 36 (33.3) | 2 (1.9) | 3 (15.0) | 0 | 4 (23.5) | 0 | 43 (29.3) | 2 (1.4) |
| Dry eye | 38 (35.2) | 0 | 1 (5.0) | 0 | 1 (5.9) | 0 | 41 (27.9) | 1 (0.7) |
| Abdominal pain | 25 (23.1) | 6 (5.6) | 4 (20.0) | 0 | 4 (23.5) | 2 (11.8) | 34 (23.1) | 8 (5.4) |
| Hypophosphatemia | 28 (25.9) | 16 (14.8) | 4 (20.0) | 3 (15.0) | 2 (11.8) | 2 (11.8) | 34 (23.1) | 21 (14.3) |
| Back pain | 27 (25.0) | 1 (0.9) | 1 (5.0) | 0 | 4 (23.5) | 3 (17.6) | 32 (21.8) | 4 (2.7) |
| Dry skin | 30 (27.8) | 1 (0.9) | 0 | 0 | 0 | 0 | 32 (21.8) | 1 (0.7) |
| Pain in extremity | 26 (24.1) | 1 (0.9) | 3 (15.0) | 2 (10.0) | 0 | 0 | 29 (19.7) | 3 (2.0) |
| Urinary tract infection | 21 (19.4) | 3 (2.8) | 2 (10.0) | 0 | 3 (17.6) | 1 (5.9) | 27 (18.4) | 4 (2.7) |
| Edema peripheral | 16 (14.8) | 1 (0.9) | 4 (20.0) | 0 | 6 (35.3) | 0 | 26 (17.7) | 1 (0.7) |
| Weight decreased | 20 (18.5) | 2 (1.9) | 4 (20.0) | 1 (5.0) | 1 (5.9) | 0 | 26 (17.7) | 3 (2.0) |
| Palmar-plantar erythrodysesthesia syndrome | 23 (21.3) | 7 (6.5) | 1 (5.0) | 0 | 0 | 0 | 24 (16.3) | 7 (4.8) |
| Headache | 20 (18.5) | 0 | 1 (5.0) | 0 | 2 (11.8) | 0 | 23 (15.6) | 0 |
| Hypercalcemia | 17 (15.7) | 2 (1.9) | 5 (25.0) | 1 (5.0) | 1 (5.9) | 0 | 23 (15.6) | 3 (2.0) |
| Dehydration | 17 (15.7) | 3 (2.8) | 1 (5.0) | 1 (5.0) | 3 (17.6) | 1 (5.9) | 22 (15.0) | 5 (3.4) |
| Pyrexia | 15 (13.9) | 1 (0.9) | 4 (20.0) | 0 | 3 (17.6) | 0 | 22 (15.0) | 1 (0.7) |
| Anemia | 16 (14.8) | 3 (2.8) | 2 (10.0) | 1 (5.0) | 3 (17.6) | 1 (5.9) | 21 (14.3) | 5 (3.4) |
| Dizziness | 19 (17.6) | 0 | 1 (5.0) | 0 | 1 (5.9) | 1 (5.9) | 21 (14.3) | 1 (0.7) |
| Asthenia | 15 (13.9) | 0 | 4 (20.0) | 1 (5.0) | 1 (5.9) | 1 (5.9) | 20 (13.6) | 2 (1.4) |
| Epistaxis | 19 (17.6) | 0 | 1 (5.0) | 0 | 0 | 0 | 20 (13.6) | 0 |
| Myalgia | 15 (13.9) | 2 (1.9) | 1 (5.0) | 0 | 2 (11.8) | 0 | 18 (12.2) | 2 (1.4) |
| Blood creatinine increased | 10 (9.3) | 1 (0.9) | 2 (10.0) | 0 | 5 (29.4) | 1 (5.9) | 17 (11.6) | 2 (1.4) |
| Dyspnea | 11 (10.2) | 2 (1.9) | 3 (15.0) | 0 | 3 (17.6) | 0 | 17 (11.6) | 2 (1.4) |
| Dyspepsia | 14 (13.0) | 0 | 2 (10.0) | 0 | 0 | 0 | 16 (10.9) | 0 |
| Gastroesophageal reflux disease | 13 (12.0) | 1 (0.9) | 1 (5.0) | 0 | 2 (11.8) | 0 | 16 (10.9) | 1 (0.7) |
| Hyponatremia | 7 (6.5) | 3 (2.8) | 5 (25.0) | 4 (20.0) | 4 (23.5) | 1 (5.9) | 16 (10.9) | 8 (5.4) |
| Abdominal pain upper | 12 (11.1) | 2 (1.9) | 2 (10.0) | 0 | 1 (5.9) | 0 | 15 (10.2) | 2 (1.4) |
| Blood ALP increased | 12 (11.1) | 3 (2.8) | 1 (5.0) | 1 (5.0) | 2 (11.8) | 1 (5.9) | 15 (10.2) | 5 (3.4) |
| Insomnia | 13 (12.0) | 0 | 2 (10.0) | 0 | 0 | 0 | 15 (10.2) | 0 |
| Pruritus | 13 (12.0) | 0 | 1 (5.0) | 0 | 1 (5.9) | 0 | 15 (10.2) | 0 |
| ALT increased | 12 (11.1) | 2 (1.9) | 0 | 0 | 1 (5.9) | 1 (5.9) | 13 (8.8) | 3 (2.0) |
| AST increased | 9 (8.3) | 3 (2.8) | 1 (5.0) | 0 | 2 (11.8) | 1 (5.9) | 12 (8.2) | 4 (2.7) |
| Hyperbilirubinemia | 9 (8.3) | 3 (2.8) | 2 (10.0) | 0 | 1 (5.9) | 0 | 12 (8.2) | 3 (2.0) |
| Hypertension | 9 (8.3) | 3 (2.8) | 3 (15.0) | 1 (5.0) | 0 | 0 | 12 (8.2) | 4 (2.7) |
| Acute kidney injury | 7 (6.5) | 2 (1.9) | 1 (5.0) | 0 | 3 (17.6) | 1 (5.9) | 11 (7.5) | 3 (2.0) |
| Hypokalemia | 9 (8.3) | 3 (2.8) | 0 | 0 | 1 (5.9) | 0 | 10 (6.8) | 3 (2.0) |
| Hypotension | 6 (5.6) | 4 (3.7) | 2 (10.0) | 2 (10.0) | 2 (11.8) | 0 | 10 (6.8) | 6 (4.1) |
| Blood bilirubin increased | 8 (7.4) | 4 (3.7) | 0 | 0 | 1 (5.9) | 0 | 9 (6.1) | 4 (2.7) |
| Cholangitis | 7 (6.5) | 4 (3.7) | 1 (5.0) | 0 | 1 (5.9) | 1 (5.9) | 9 (6.1) | 5 (3.4) |
| Ascites | 5 (4.6) | 2 (1.9) | 2 (10.0) | 1 (5.0) | 0 | 0 | 7 (4.8) | 3 (2.0) |
| Pleural effusion | 3 (2.8) | 1 (0.9) | 2 (10.0) | 2 (10.0) | 1 (5.9) | 1 (5.9) | 6 (4.1) | 4 (2.7) |
| Pneumonia | 3 (2.8) | 2 (1.9) | 1 (5.0) | 0 | 1 (5.9) | 1 (5.9) | 5 (3.4) | 3 (2.0) |
| Failure to thrive | 2 (1.9) | 2 (1.9) | 0 | 0 | 1 (5.9) | 1 (5.9) | 3 (2.0) | 3 (2.0) |
| Sepsis | 2 (1.9) | 2 (1.9) | 1 (5.0) | 1 (5.0) | 0 | 0 | 3 (2.0) | 3 (2.0) |
| Small intestine obstruction | 2 (1.9) | 2 (1.9) | 0 | 0 | 1 (5.9) | 1 (5.9) | 3 (2.0) | 3 (2.0) |

ALP, alkaline phosphatase; ALT, alanine aminotransferase; AST, aspartate aminotransferase; FGF, fibroblast growth factor; FGFR, FGF receptor; TEAE, treatment-emergent adverse event.
^a^ Total number includes 2 patients who did not have confirmed *FGF*/*FGFR* status by central laboratory testing and were not assigned to any cohort.

^b^ All any-grade TEAEs occurring in ≥10% and grade ≥3 TEAEs occurring in ≥2% of the total population are shown.

# Figure S1. Patient Disposition

Enrolled (n=147)

**Cohort A
*FGFR2* rearrangement or fusion
n=108**

**Cohort B
Other *FGF/FGFR* alteration
n=20**

**Cohort C**

No *FGF/FGFR* alteration
n=17

Undetermined *FGF/FGFR* alteration
n=2

Received pemigatinib
n=108

Received pemigatinib
n=20

Received pemigatinib
n=17

Received pemigatinib
n=2

Analyzed for efficacy and safety
n=108

Analyzed for efficacy and safety

n=20

Analyzed for efficacy and safety

n=17

Analyzed for safety but not efficacy

n=2

Discontinued treatment, n=106 (98.1%)

- Progressive disease, n=77 (71.3%)
- Withdrawal by patient, n=8 (7.4%)
- Physician decision, n=7 (6.5%)
- Adverse event, n=6 (5.6%)
- Study terminated by sponsor,
  n=2 (1.9%)
- Death, n=1 (0.9%)
- Other, n=5 (4.6%)

Discontinued treatment, n=20 (100.0%)

- Progressive disease, n=15 (75.0%)
- Adverse event, n=2 (10.0%)
- Withdrawal by patient, n=2 (10.0%)
- Physician decision, n=1 (5.0%)

Discontinued treatment, n=17 (100.0%)

- Progressive disease, n=11 (64.7%)
- Adverse event, n=2 (11.8%)
- Withdrawal by patient, n=2 (11.8%)
- Lost to follow-up, n=1 (5.9%)
- Other, n=1 (5.9%)

Discontinued treatment, n=2 (100.0%)

- Progressive disease, n=2 (100.0%)

Assessed for eligibility (n=171)

Centrally prescreened for *FGF/FGFR* status (n=1206)^a^

*FGF/FGFR report in hand (n=85)^b^*

Patients were assigned to 1 of 3 cohorts based on *FGF/FGFR* alteration status. Patients whose tumor samples could not be analyzed for *FGF/FGFR* status by the central laboratory were assigned “Undetermined” and were not included in the efficacy analysis.

FGF, fibroblast growth factor; FGFR, FGF receptor.

^a^ FoundationOne^®^, Foundation Medicine.

^b^ Most patients with report in hand had undergone FoundationOne^®^ testing for *FGF/FGFR* status.

Figure S2. Subgroup Analysis of ORR Based on IRC Assessment According to RECIST v1.1 in Cohort A (*FGFR2* Fusions or Rearrangements; Efficacy-Evaluable Population)


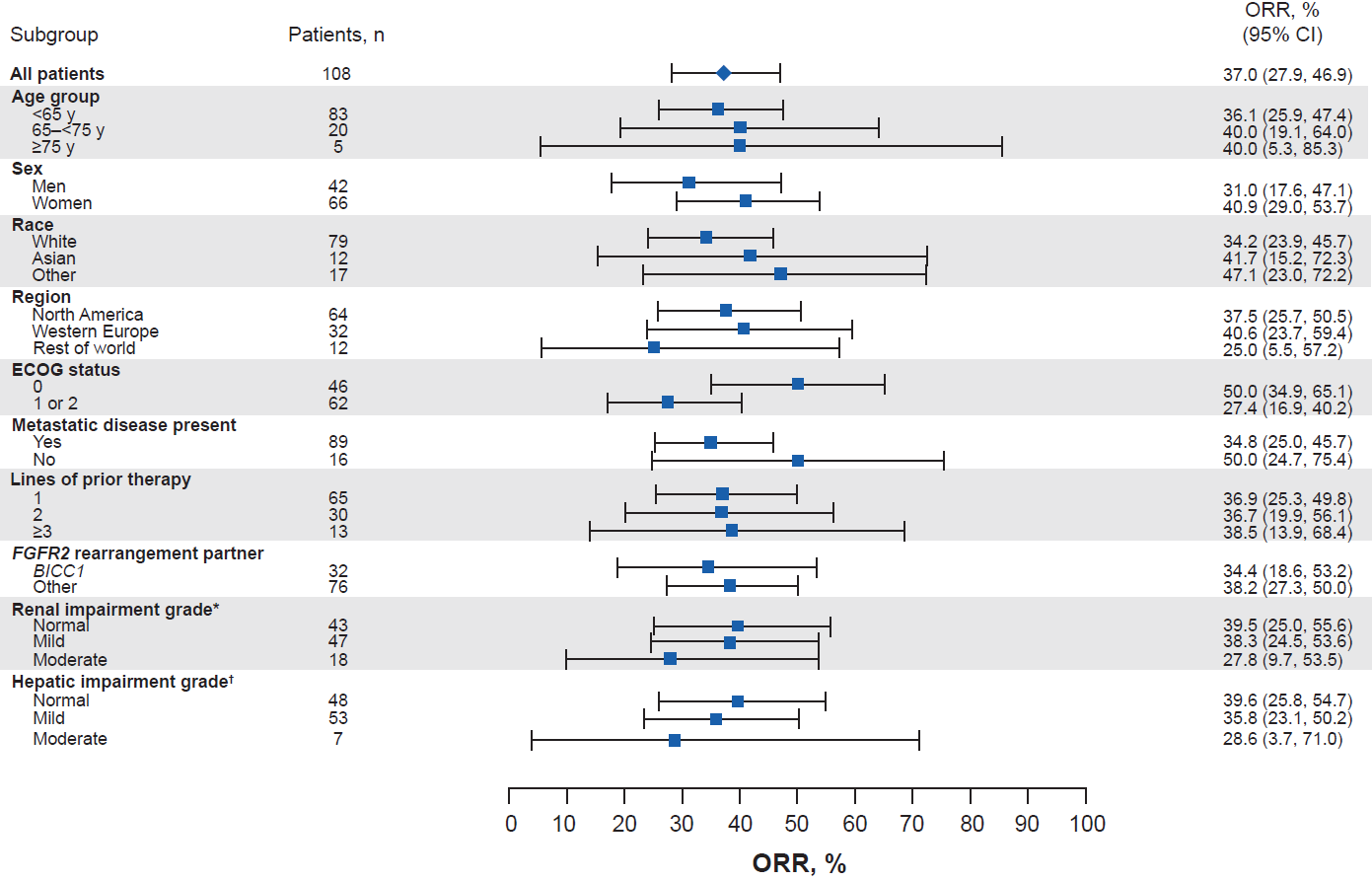


ALT, alanine aminotransferase; AST, aspartate aminotransferase; ECOG, Eastern Cooperative Oncology Group; eGFR, estimated glomerular filtration rate; FGFR, fibroblast growth factor receptor; IRC, independent review committee; MDRD, Modification of Diet in Renal Disease; NCI, National Cancer Institute; ORR, objective response rate; RECIST, Response Evaluation Criteria in Solid Tumors; ULN, upper limit of normal.

^a^ Baseline renal impairment grade based on eGFR calculated using the MDRD equation: normal, eGFR ≥90 mL/min/1.73 m^2^; mild, eGFR ≥60 and <90 mL/min/1.73 m^2^; moderate, eGFR ≥30 to <60 mL/min/1.73 m^2^.

^b^ Degree of hepatic impairment based on NCI Hepatic Working Group Criteria: mild, total bilirubin ≤1.5× ULN and ALT or AST >ULN; moderate, >1.5–3× ULN and any ALT or AST.

Figure S3. Kaplan-Meier Estimate of DOR Based on IRC Assessment for Cohort A (*FGFR2* Rearrangements or Fusions; Efficacy-Evaluable Population)


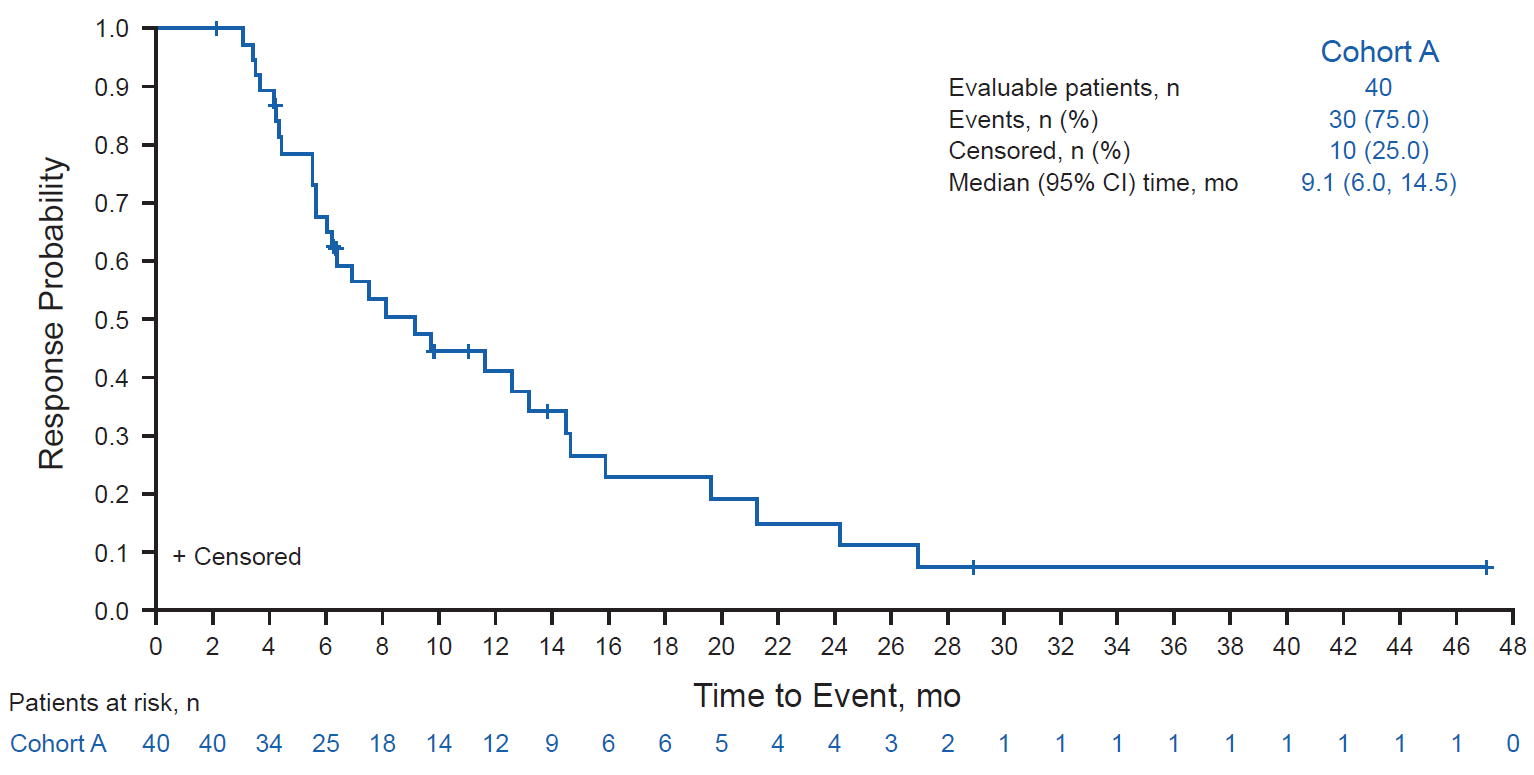


DOR, duration of response; FGFR, fibroblast growth factor receptor; IRC, independent review committee.

Figure S4. Best Percentage Change From Baseline in Target Lesion Size Based on IRC Assessment Among Efficacy-Evaluable Patients in (A) Cohort B (Other *FGF*/*FGFR* Alterations) and (B) Cohort C (no *FGF/FGFR* alterations)

**A**


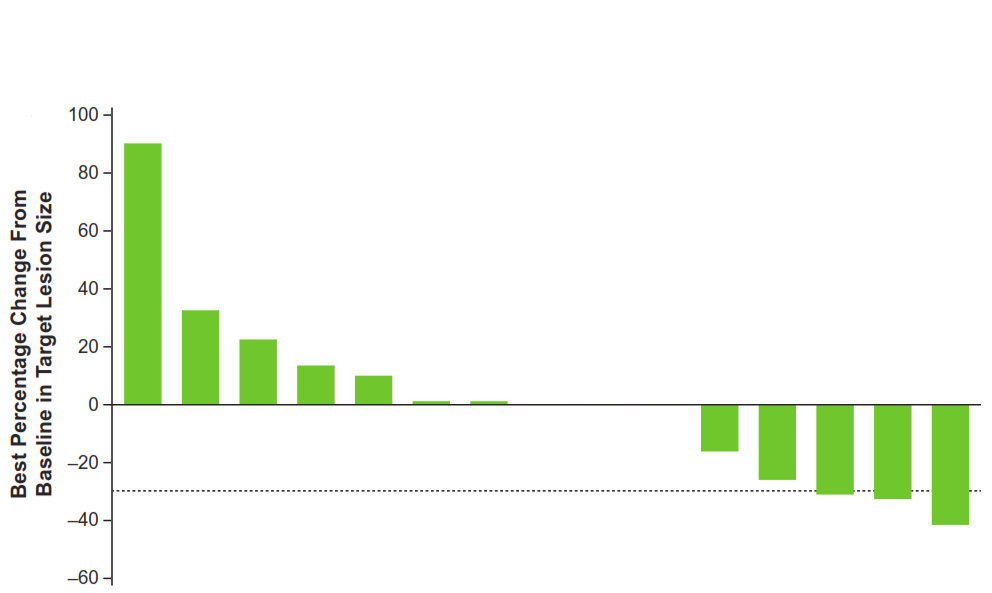


**B**


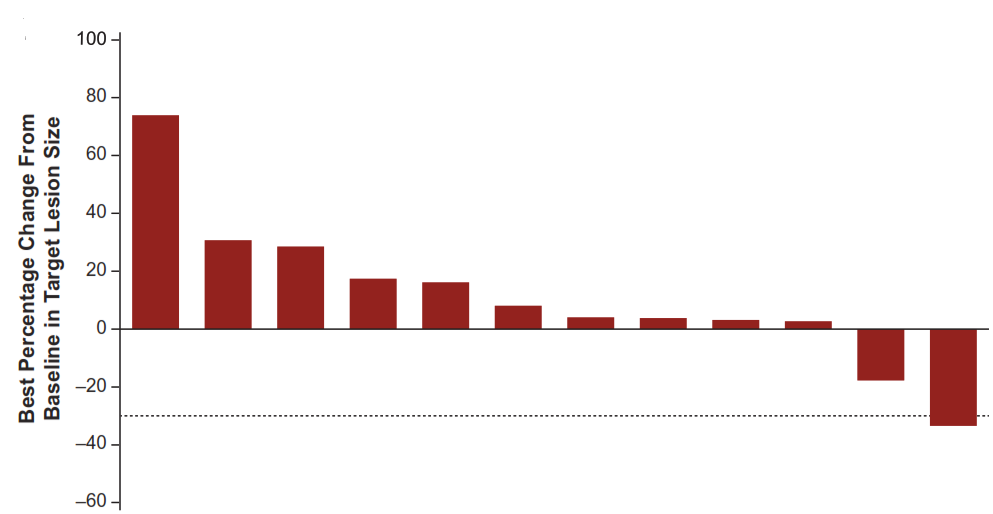


FGF, fibroblast growth factor; FGFR, FGF receptor; IRC, independent review committee.

Figure S5. Subgroup Analysis of PFS Based on IRC Assessment in Cohort A (*FGFR2* Fusions or Rearrangements; Efficacy-Evaluable Population)


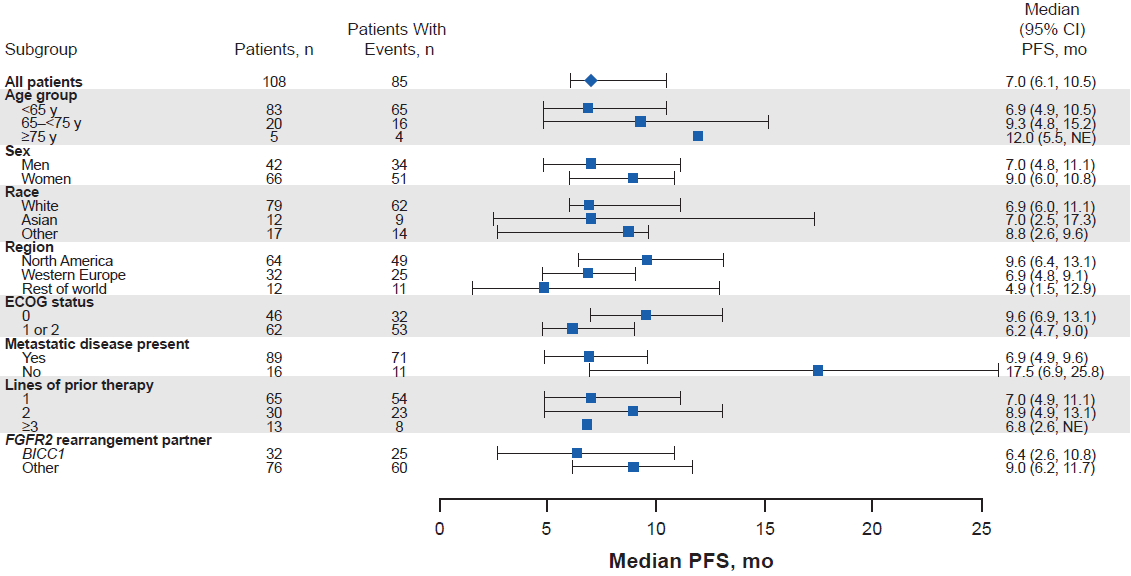


ECOG, Eastern Cooperative Oncology Group; FGFR, fibroblast growth factor receptor; IRC, independent review committee; NE, not estimable; PFS, progression-free survival.

Figure S6. Subgroup Analysis of OS in Cohort A (*FGFR2* Fusions or Rearrangements; Efficacy-Evaluable Population)


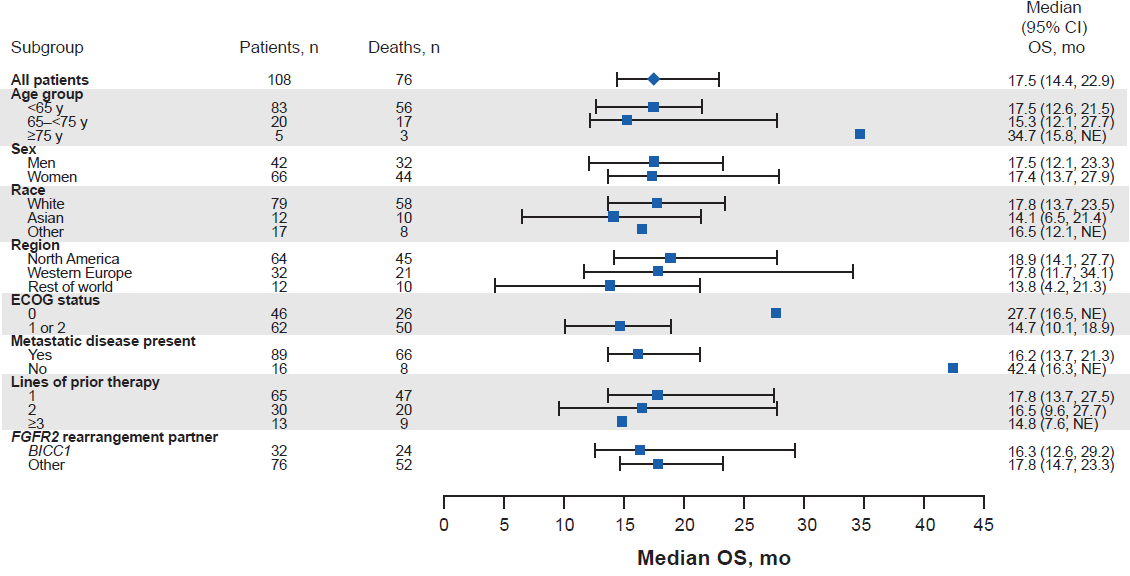


ECOG, Eastern Cooperative Oncology Group; FGFR, fibroblast growth factor receptor; OS, overall survival; NE, not estimable.
